# The *Streptococcus agalactiae* LytSR two-component regulatory system promotes vaginal colonization and virulence *in vivo*

**DOI:** 10.1128/spectrum.01970-24

**Published:** 2024-10-14

**Authors:** Hajar AlQadeeb, Murielle Baltazar, Adrian Cazares, Tiraput Poonpanichakul, Morten Kjos, Neil French, Aras Kadioglu, Marie O’Brien

**Affiliations:** 1 Department of Medical Laboratory, College of Applied Medical Sciences in Al-Kharj, Prince Sattam Bin Abdulaziz University, Al-Kharj, Saudi Arabia; 2 Department of Clinical Infection, Microbiology and Immunology, University of Liverpool, Liverpool, United Kingdom; 3 Parasites and Microbes Programme, Wellcome Sanger Institute, Hinxton, United Kingdom; 4 Chakri Naruebodindra Medical Institute, Faculty of Medicine Ramathibodi Hospital, Mahidol University, Nakhon Pathom, Thailand; 5 Faculty of Chemistry, Biotechnology and Food Science, Norwegian University of Life Sciences, Ås, Norway; 6 ReNewVax Ltd, Liverpool, United Kingdom; Michigan State University, East Lansing, Michigan, USA

**Keywords:** *Streptococcus agalactiae*, group B *Streptococcus*, newborn infections, LytSR two-component regulatory system, CC-17, pathogenesis

## Abstract

*Streptococcus agalactiae* (or group B *Streptococcus*, GBS) is a leading cause of neonatal sepsis and meningitis globally. To sense and respond to variations in its environment, GBS possesses multiple two-component regulatory systems (TCSs), such as LytSR. Here, we aimed to investigate the role of LytSR in GBS pathogenicity. We generated an isogenic *lytS* knockout mutant in a clinical GBS isolate and used a combination of phenotypic *in vitro* assays and *in vivo* murine models to investigate the contribution of *lytS* to the colonization and invasive properties of GBS. Deletion of the *lytS* gene in the GBS chromosome resulted in significantly higher survival rates in mice during sepsis, accompanied by reduced bacterial loads in blood, lung, spleen, kidney, and brain tissues compared to infection with the wild-type strain. In a mouse model of GBS vaginal colonization, we also observed that the *lytS* knockout mutant was cleared more readily from the vaginal tract compared to its wild-type counterpart. Interestingly, lower levels of proinflammatory cytokines were found in the serum of mice infected with the *lytS* mutant. Our results demonstrate that the LytSR TCS plays a key role in GBS tissue invasion and pathogenesis, and persistence of mucosal colonization.

**IMPORTANCE:**

*Streptococcus agalactiae (*group B *Streptococcus,* or GBS) is a common commensal of the female urogenital tract and one of WHO’s priority pathogens. The bacterium has evolved mechanisms to adapt and survive in its host, many of which are regulated via two-component signal transduction systems (TCSs); however, the exact contributions of TCSs toward GBS pathogenicity remain largely obscure. We have constructed a TCS *lytS-*deficient mutant in a CC-17 hypervirulent GBS clinical isolate. Using murine models, we showed that LytSR regulatory system is essential for vaginal colonization via promoting biofilm production. We also observed that *lytS* deficiency led to significantly attenuated virulence properties and lower levels of proinflammatory cytokines in blood. Our findings are of significant importance in that they unveil a previously unreported role for LytSR in GBS and pave the way toward a better understanding of its ability to transition from an innocuous commensal to a deadly pathogen.

## INTRODUCTION

*Streptococcus agalactiae*, commonly known as group B *Streptococcus* (GBS), is an opportunistic pathogen that commonly colonizes the gastrointestinal and genitourinary tracts of healthy adults, the elderly, and pregnant women (1–3). Under given circumstances, GBS can cause life-threatening invasive diseases, such as pneumonia, sepsis, and meningitis, especially in vulnerable groups such as neonates (1). To date, 10 GBS serotypes have been described based on the type of capsular polysaccharide, with serotypes Ia, Ib, II, III, and V being the most common causes of life-threatening and self-limiting infections (4). Multilocus sequence typing system was developed to further classify GBS into clonal complexes (CCs) (5). Certain CCs were found to be commonly associated with invasive disease, e.g., CC-17, while others are mainly presented as colonizing strains, e.g., CC-1 and CC-19 (5, 6). In a recent report, the genomic sequence analysis of nearly 2,000 GBS carriage and invasive disease-associated isolates originating from different hosts (human and animal) and five different countries led to the identification of 100 accessory and core genes associated with the hypervirulent CC-17. Among them, the proton-dependent oligopeptide transporter family and iron complex transport system permease protein FeuC were deemed particularly important (7).

GBS possesses a number of virulence factors, including adhesins such as Srr1/Srr2, the fibrinogen-binding proteins (FbsA, FbsB, and FbsC), the laminin-binding protein (Lmb), the surface-associated serine protease ScpB or C5a peptidase, the plasminogen-binding surface protein (PbsP), the pili (proteins PilA, PilB, and PilC), the GBS immunogenic bacterial adhesin (BibA), and the hyaluronidase HylB [reviewed in references (8, 9)]. The polysaccharide capsule encoded by the *cps* operon participates in GBS resistance to complement deposition, opsonization, and phagocytosis (10, 11), while the β-hemolysin/cytolysin, also referred as the “hemolytic pigment” or granadaene, is responsible for the hemolytic activity of GBS and is encoded by the *cyl* operon (12, 13). In CC-17, in particular, the adhesins Srr2 and HvgA, and the pilus protein PI-2b are known to play an important role in host cell adhesion, colonization, and tolerance to phagocytosis (14–16). While all these GBS virulence factors are well documented, their genetic regulation remains largely unexplored.

Of the regulatory factors known to be essential (6), two-component regulatory systems (TCSs) are major contributors to bacterial responses to environmental changes through the regulation of gene expression in response to stimuli (17, 18). TCSs consist of a sensor histidine kinase, which once activated by an external signal or stress conditions, autophosphorylates its cytoplasmic domain and relays the phospho-group to the transcriptional response regulator that in turn regulates the expression of target genes (18). The size and complexity of the regulons for TCS are highly variable, ranging from a few genes to hundreds of genes (19). To date, around 20 TCSs have been described in GBS, some of which have been shown to play important roles in bacterial pathogenesis (20). Examples include the CovRS, in which deletion mutants have shown different effects on virulence depending on the infection model used (20–22), and HssRS, a regulator of heme transport which has been shown to be critical during systemic infection in a mouse model (23). Furthermore, a number of TCSs in GBS are known to be important for the regulation of colonization, adhesion, and resistance to antimicrobials (20). Genome analysis of *S. agalactiae* has also identified orthologs of TCSs known to participate in the virulence in other species; however, their role in *S. agalactiae* is yet unknown. One such example is LytSR (24).

The LytSR two-component system is composed of the sensor LytS and the regulator LytR. LytSR is widely conserved across species and has been shown to be important for virulence-associated phenotypes, such as biofilm formation and resistance against host cationic antimicrobial peptides (CAMPs) (25). In different staphylococcal species, lack of LytSR has been shown to alter biofilm formation capabilities potentially by altered autolysis (24, 26, 27), while the deletion of *lytS* led to a significantly increased susceptibility to membrane-damaging CAMPs in both *in vitro* and *in vivo S. aureus* infection models (25). The direct regulatory function of LytSR has been linked to cell autolysis and metabolism in different species, as the LytSR system has been shown to sense both changes in membrane potential and extracellular metabolic signals, such as pyruvate, glucose, and oxygen (28). From studies in *S. aureus*, *Staphylococcus epidermidis*, *Streptococcus mutans,* and *Bacillus subtilis,* it is established that LytSR controls the expression of the downstream *lrgAB* operon which encodes a pore-forming holin (29). The actual role of LrgAB is not fully established; it may be important for the regulation of murein hydrolase activity similar to other well-characterized holins, but recent research has also indicated that LrgAB is transporting metabolic by-products such as pyruvate (29, 30). Regulation of LrgAB by LytSR could thus also be used by the bacteria to integrate extracellular signals with the metabolic state of the cell (28). Furthermore, transcriptional analyses have indicated that LytSR have a global regulatory role. Microarray analysis in *S. aureus* revealed that removal of *lytSR* affected the expression of 267 genes encoding proteins involved in a variety of functions, such as carbohydrate, energy, and nucleotide metabolism (27). Similarly, the transcriptional analysis of *lytSR* mutants in *S. mutans* and *S. epidermidis* showed equivalent effects on gene expression (31, 32). However, the role of LytSR two-component system in GBS pathogenesis remains largely unknown. Here, we aimed to investigate the impact of *lytS* deletion *in vitro* on GBS autolysis, hemolysis, cell adhesion, and biofilm formation as well as *in vivo* on mucosal colonization and invasive properties.

## RESULTS

### Phenotypic characterization of *lytS* deletion in GBS

**Fig 1:**
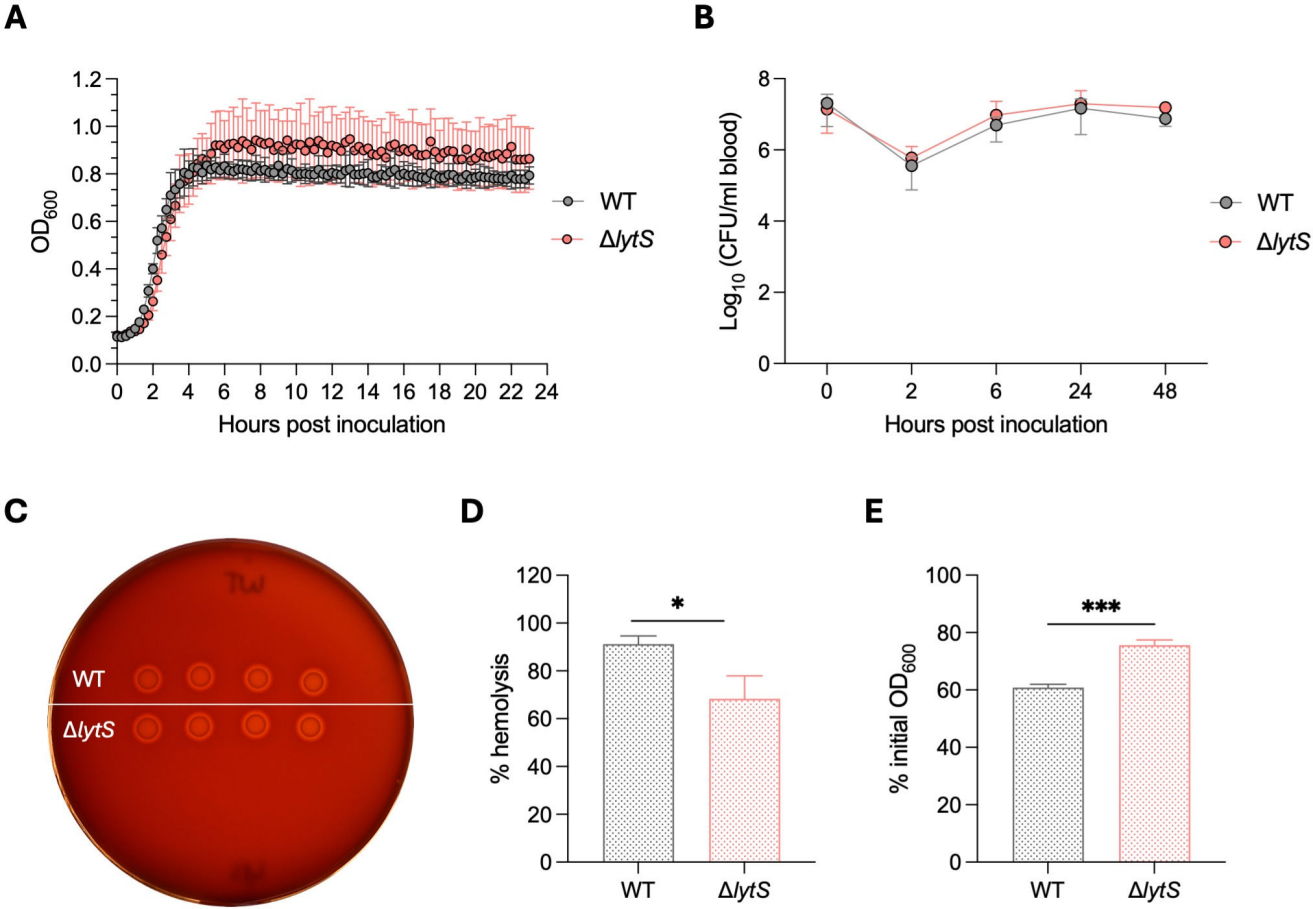
Phenotypic characterization of GBS wild-type and the isogenic Δ*lytS* strains. (**A**) Bacterial growth of GBS strain HQ199 WT and the isogenic Δ*lytS* mutant was determined in Todd-Hewitt broth at 37°C and 5% CO_2_. Optical density at 600 nm (OD_600_) was measured for 24 hours. Data represent the mean ± SD of three independent experiments. (**B**) Bacterial survival in blood. Bacterial strains were grown in human whole blood at 37°C for 48 hours, and viable bacteria were enumerated. Data represent the mean ± SD of three independent experiments. (**C**) Observation of β-hemolysis on sheep blood agar base medium. Bacteria were grown at 37°C overnight. (**D**) Hemolytic activity. Bacterial strains were incubated in fresh sheep red blood cells. After incubation for 30 min, samples were centrifuged, and the levels of hemoglobin released in the supernatant were determined by measuring the optical density at OD_420_. Data represent the mean ± SD of three independent experiments. (**E**) Rates of bacterial autolysis. Bacterial strains were grown at an OD_600_ = 1 prior to incubation in 0.01% Triton-X-100 at 37°C with shaking. After incubation for 120 min, OD_600_ was measured and converted as the percent of the initial OD_600_ reading. Data represent the mean ± SD of three independent experiments. Statistical analysis was performed using the unpaired *t* test. *P < 0.05 and ***P < 0.001.

To investigate the contribution of LytSR in GBS virulence, we deleted the *lytS* gene (encoding the sensor of the system) in a serotype III CC-17 clinical isolate (strain HQ199) to generate the isogenic *lytS*-deficient strain HQ199Δ*lytS*. We first compared the growth of the Δ*lytS* mutant to its wild-type (WT) counterpart in planktonic cultures *in vitro*. Both strains showed similar growth over 24 hours in Todd-Hewitt broth (THB; Fig. 1A). We also monitored the survival of both strains in human whole blood, this experimental setup serving as an *ex vivo* assay to mimic the *in vivo* blood environment. Both WT and Δ*lytS* isolates showed similar survival rates in human whole blood over a 48-hour period (Fig. 1B). These results indicate that the absence of the *lytS* gene did not affect bacterial growth or survival *in vitro*.

Next, we analyzed the hemolytic activity of both WT and Δ*lytS* strains. Macroscopic observation on blood agar base (BAB) medium showed similar β-hemolysis for both isolates (Fig. 1C); however, quantification of hemolysis showed significantly decreased hemolytic activity in the *lytS* mutant compared to the WT strain (Fig. 1D), suggesting that *lytS* deficiency impaired the ability of GBS to lyse red blood cells (RBCs). Similarly, we investigated the impact of *lytS* deficiency on GBS autolysis. Triton X-100-induced autolysis assay showed that the Δ*lytS* mutant displayed a significantly lower rate of autolysis compared to the WT strain (Fig. 1E; Fig. S1), which suggests that *lytS* deficiency impaired autolysis in GBS.

### *lytS* deficiency alters GBS colonization in the genital tract

**Fig 2:**
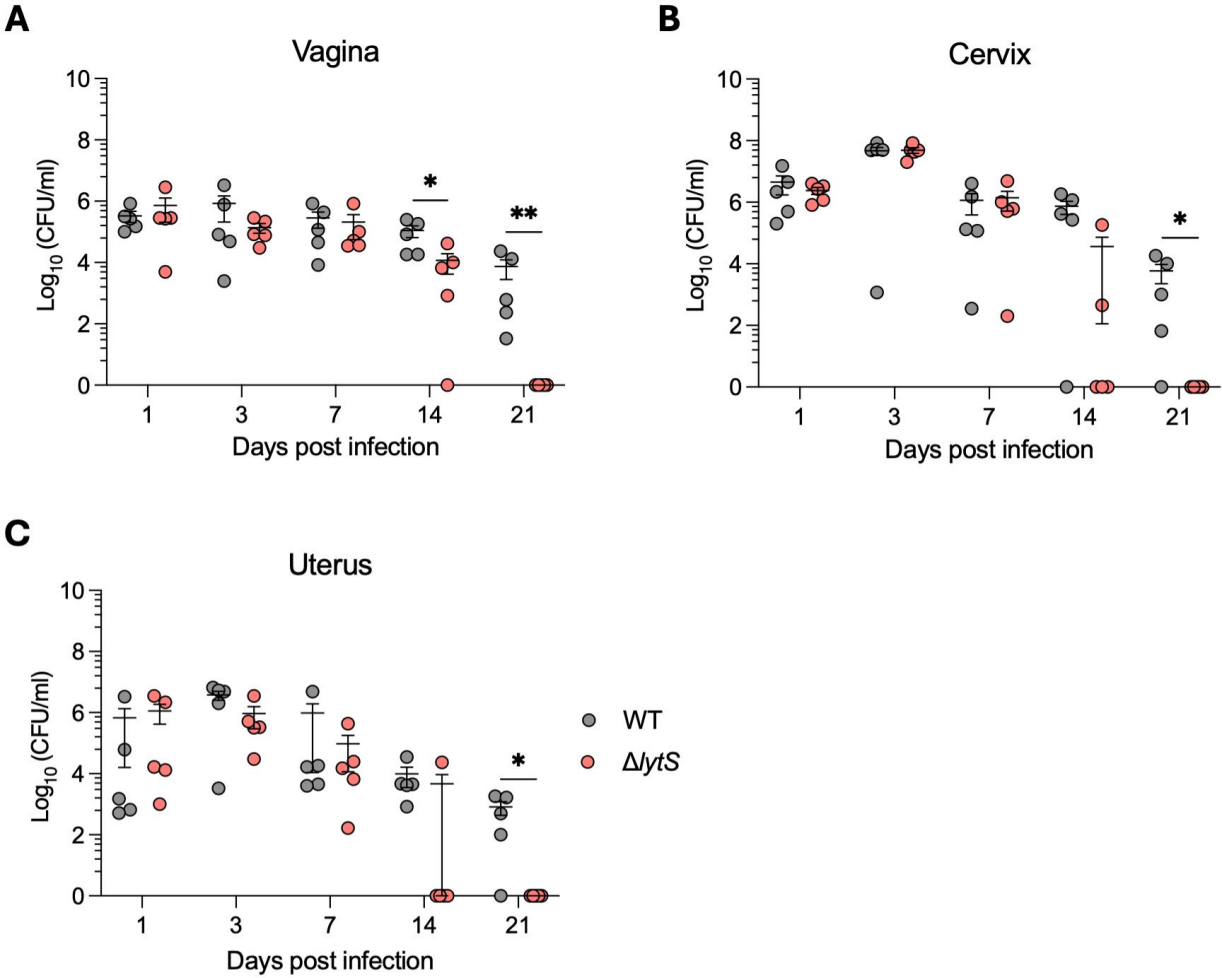
*lytS* deficiency impairs GBS colonization and persistence in the genital tract of mice. Female Balb/c mice (*n* = 30 per group, 5 mice per day) were infected intravaginally with 1 × 10^6^ CFU of GBS HQ199 WT or the isogenic Δ*lytS* strains. Levels of bacterial colonization in the vagina (**A**), cervix (**B**), and uterus (**C**) were quantified at days 1, 3, 7, 14, and 21 post infection. Each dot represents a single mouse, and error bars represent the mean ± SEM in each time point. Statistical analysis between the two groups in each day was performed using the Mann-Whitney *U* test. *P < 0.05 and **P < 0.01.

The genital tract is a common tissue niche for GBS colonization (33). Hence, we explored whether and how *lytS* deficiency impacted on vaginal colonization *in vivo*. One day prior to infection with GBS, female Balb/c mice were treated with 17β-estradiol hormone to mimic the estrus cycle (34). The following day, mice were intravaginally colonized with 1 × 10^6^ CFU of GBS WT or Δ*lytS* strains, and the level of bacterial colonization was quantified in vaginal, cervical, and uterine tissues over a 21-day period. Both WT and Δ*lytS* strains established stably and at equivalent levels in the vagina and cervix of mice during the first 7 days following infection (Fig. 2A and B), and both isolates were also detectable in the uterus at similar levels since day 1 post infection (Fig. 2C), suggesting that both GBS isolates migrated rapidly from the vagina to the uterus. Interestingly, at day 14 post infection, mice infected with the Δ*lytS* mutant displayed significantly lower CFU counts in the vagina (*P* = 0.03), while several mice showed no bacteria in cervix or uterus tissues. A total clearance of the mutant strain was observed in all tissues at day 21 post infection (Fig. 2). Conversely, colonization with the WT strain remained stable and persisted at a density of 10^4^ CFU/mL of tissue in the vagina, cervix, and uterus up to day 21 post infection (Fig. 2). These results show that the absence of *lytS* resulted in reduced vaginal colonization and promoted a more rapid clearance of GBS from the genital tract.

We next examined whether the impaired ability of the Δ*lytS* mutant to colonize the genital tract of mice was due to an alteration of its capability to adhere and/or invade the vaginal epithelium and produce biofilm. We performed *in vitro* adherence and invasion assays using the human vaginal epithelial VK2/E6E7 cell line. The Δ*lytS* mutant showed slightly reduced adhesion and invasion to epithelial cells compared to its WT counterpart (Fig. S2A and B), albeit this was not statistically significant. In addition, the Δ*lytS* mutant produced significantly less biofilm than the WT strain *in vitro* (*P* < 0.01) (Fig. S2C). Taken together, these results suggest that *lytS* deficiency impaired the ability of GBS to colonize the genital tract of mice by reducing its biofilm production and altering its cell adhesion and invasion properties.

### LytSR plays a key role in GBS virulence and dissemination *in vivo*

**Fig 3:**
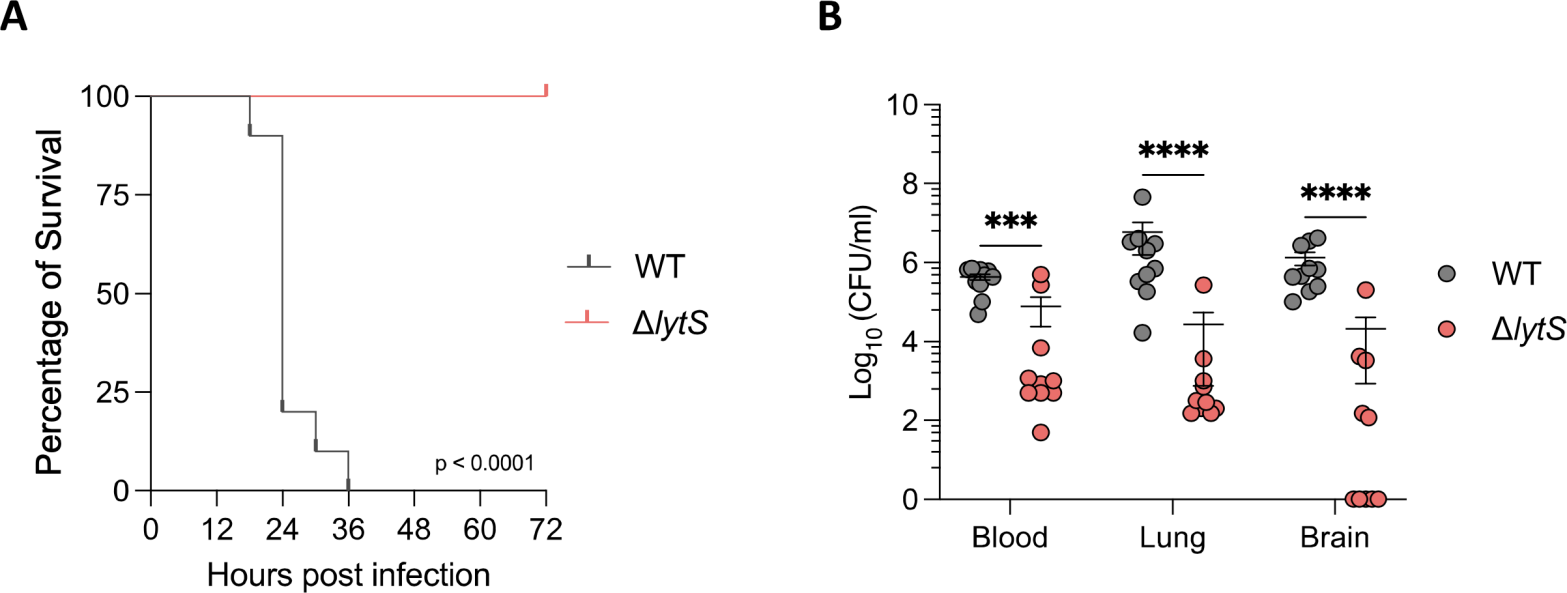
*lytS* deficiency reduces GBS virulence and dissemination in a mouse sepsis model. (**A**) Kaplan-Meier curve representing percentage of survival of female Balb/c mice (*n* = 10 per group) infected intravenously with 1 × 10^8^ CFU of GBS HQ199 wild-type or the isogenic Δ*lytS* strains. Statistical analysis was performed using the log-rank (Mantel-Cox) test. (**B**) Levels of bacterial loads in blood, lung, and brain tissues at time of death. In the WT group, animals were euthanized upon reaching humane endpoints at 18, 24, 30, and 36 hours post infection (*n* = 1, 7, 1, and 1, respectively). In the Δ*lytS* group, all tissues were collected at 72 hours post infection (*n* = 10). Each dot represents a single mouse, and error bars represent the mean ± SEM. Statistical analysis was performed using the Mann-Whitney *U* test. ***P < 0.001 and ****P < 0.0001.

We next sought whether the deletion of *lytS* contributes to GBS virulence and pathogenesis *in vivo* using a mouse sepsis model of GBS infection. All female Balb/c mice intravenously infected with the WT strain displayed 100% mortality within 36 hours of infection, while all mice infected with the Δ*lytS* mutant survived the infection (Fig. 3A). The bacterial density in tissue quantified at time of death showed that infection with the Δ*lytS* mutant was characterized by significantly reduced bacterial loads in blood, lung, and brain tissues compared to mice infected with the WT parent strain (Fig. 3B). These results suggest that the LytSR TCS played a key role in GBS virulence during sepsis.

**Fig 4:**
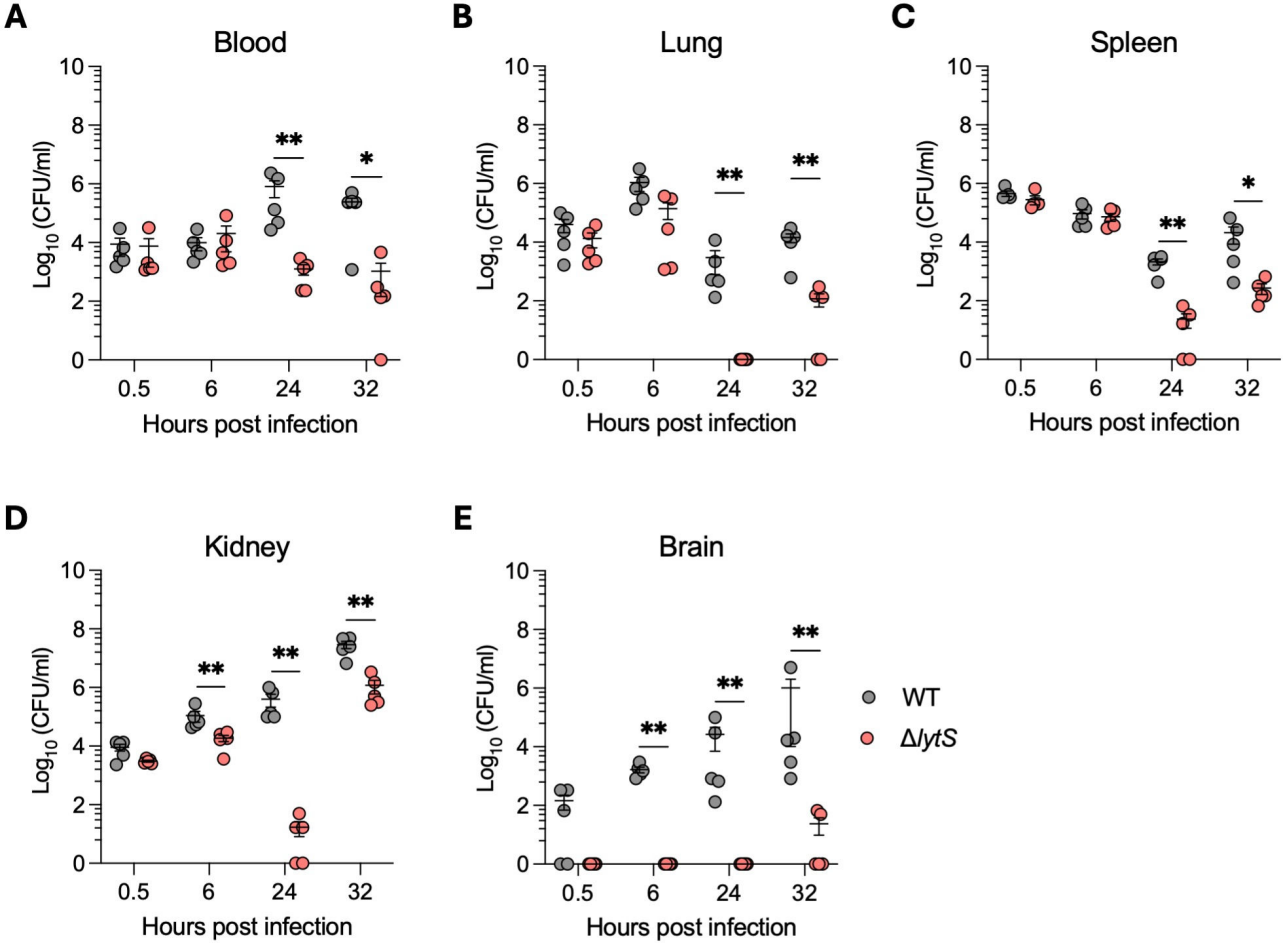
*lytS* deficiency prevents bacterial dissemination and brain invasion *in vivo* in a mouse sepsis model. Female Balb/c mice (*n* = 20 per group, 5 mice per day) were intravenously infected with 1 × 10^8^ CFU of GBS HQ199 wild-type or the isogenic Δ*lytS* strains. Levels of bacterial loads in blood (**A**), lung (**B**), spleen (**C**), kidney (**D**), and brain (**E**) tissues were quantified at 0.5, 6, 24, and 32 hours post infection. Each dot represents a single mouse, and error bars represent the mean ± SEM in each time point. Statistical analysis between the two groups in each time point was performed using the Mann-Whitney *U* test. *P < 0.05 and **P < 0.01.

To further investigate this, we next analyzed the kinetics of dissemination of both mutant and WT isolates during sepsis. Following intravenous administration of bacteria, both strains colonized blood at equivalent levels (30 min post-challenge; Fig. 4A). However, between 24 and 32 hours post infection, the bacterial burden in Δ*lytS-*infected mice significantly decreased in blood, and all tissues compared to mice were infected with the WT isolate (Fig. 4A through C). In the kidney, a significantly lower bacterial density was observed in Δ*lytS*-infected mice as early as 6 hours post infection, and this pattern persisted until 32 hours post infection (Fig. 4D). The largest differences were observed in brain tissue where WT-infected animals showed a rapid and gradual increase in CFU counts, going from 10^2^ CFU/mL of tissue at 30 min post infection to 10^6^ CFU/mL of tissue at 32 hours post infection (Fig. 4E). In contrast, no viable bacteria were detected in the brain of mice infected with the Δ*lytS* mutant until 32 hours post infection, where two mice were colonized at a density inferior to 10^2^ CFU/mL of tissue (Fig. 4E). Altogether, these results indicate that *lytS* deficiency impaired the dissemination of GBS from blood to other tissues, especially to the brain, and resulted in a more rapid bacterial clearance.

### *lytS* deficiency results in attenuated host inflammatory responses

**Fig 5:**
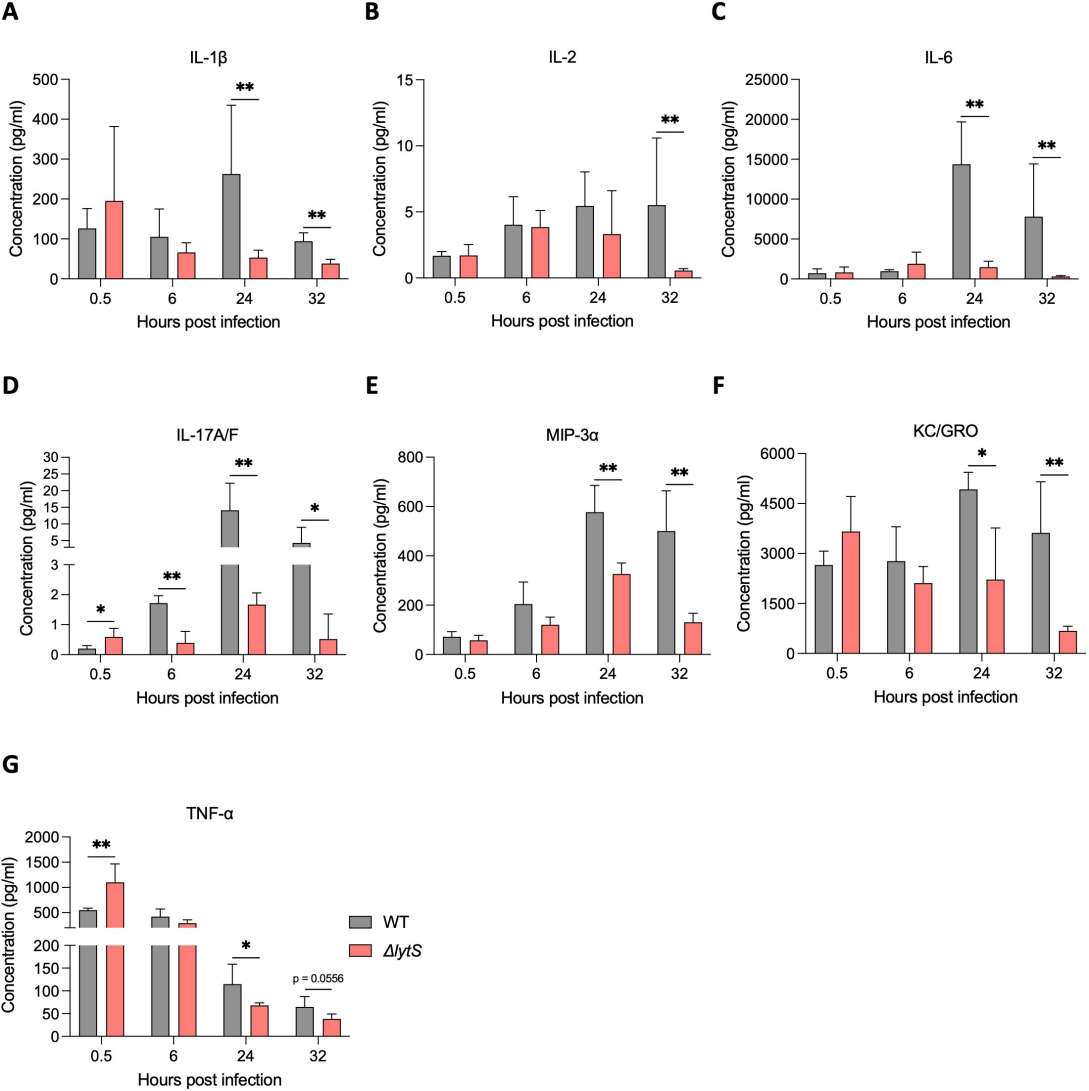
*lytS* deficiency impacts proinflammatory host responses during GBS sepsis. Female Balb/c mice (*n* = 20 per group, 5 mice per day) were intravenously infected with 1 × 10^8^ CFU of GBS HQ199 wild-type or the isogenic Δ*lytS* strains. Levels of proinflammatory cytokines IL-1β (**A**), IL-2 (**B**), IL-6 (**C**), IL-17A/F (**D**), MIP-3α (**E**), KC/GRO (**F**), and TNF-α (**G**) were quantified in the serum of mice at 0.5, 6, 24, and 32 hours post infection. Data represent the mean ± SD in each time point. Statistical analysis between the two groups in each time point was performed using the Mann-Whitney *U* test. *P < 0.05 and **P < 0.01.

As the absence of LytSR resulted in significant attenuation of GBS virulence, we examined the host immune response during sepsis by quantifying the levels of the proinflammatory cytokines IL-1β, IL-2, IL-6, IL-17A/F, MIP-3α, KC/GRO, and TNF-α in the serum of mice infected with the WT or Δ*lytS* strains. Up to 6 hours post infection, no differences were observed between the two groups of mice. At 24 and 32 hours post infection, we measured significantly lower levels of IL-1β, IL-6, MIP-3α, and KC/GRO in mice infected with the Δ*lytS* mutant as well as, for IL-2 cytokine, at 32 hours post infection (Fig. 5A through C, E and F). Transient higher levels of IL-17A/F and TNF-α were observed at 30 min post infection in the Δ*lytS* mutant group, but at 24 and 32 hours post infection, levels of both cytokines were found to be significantly lower compared to the WT-infected animals (Fig. 5D and G). Interestingly, differences in cytokine patterns closely coincided with the kinetics of bacterial loads observed in tissues (Fig. 4). Altogether, these results indicate that attenuation of GBS virulence due to the absence of LytSR also reduced its ability to induce host inflammatory responses during infection.

## DISCUSSION

*S. agalactiae* is a natural commensal of the genitourinary and gastrointestinal tracts, yet it is known to be an important cause of invasive disease in neonates and adults. Around 20 TCSs are encoded in GBS, which represents almost twice as many as other related streptococcal species such as *S. pneumoniae* or *S. pyogenes*, both of which possess only 13 TCSs within their entire genome (20, 35–37). This suggests that GBS has a strong and finely tuned capacity for sensing and responding to multiple environmental stimuli (20). The LytSR TCS was first identified in GBS in 2002 (17). However, to the best of our knowledge, its role in GBS virulence and pathogenesis has never been explored experimentally in clinically relevant models. Here we show, using an isogenic *lytS*-deficient mutant (where *lytS* encodes the sensor histidine kinase of the TCS), that LytSR has a major role to play during GBS colonization and pathogenesis *in vivo*.

Our results show that the LytSR TCS plays a significant role in GBS virulence during sepsis, whereby all mice infected with the WT strain succumbed to infection within 36 hours post infection, while all animals infected with the *lytS* mutant survived infection. Interestingly, the *lytS-*deficient strain showed a decrease in hemolytic activity, a feature that may have contributed to its reduced virulence (38–41). Infection with the Δ*lytS* mutant was associated with a significant reduction of viable bacteria in blood, lung, spleen, kidney, and, most dramatically, in the brain. Thus, LytSR would seem to be involved in *S. agalactiae* survival and proliferation in blood and tissue during different infection stages due to reduced bacterial survival in the absence of *lytS in vivo*. Notably, the dramatic reduction of bacterial load in the brain (Fig. 4E) suggests that the Δ*lytS* mutant may have a reduced capacity to cross the blood-brain barrier (BBB). Proinflammatory cytokines are known to disrupt tight junctions and increase permeability to pathogens at the BBB (42–44). Barichello et al. showed that levels of CINC-1 (also known as KC/GRO or CXCL1), IL-1β, IL-6, and TNF-α were increased in the hippocampus of neonate rats during GBS meningitis in association with BBB breakdown (45). Cytokines IL-2, IL-17A, and the chemokine MIP-3α (CCL20) have also been shown to be associated with bacterial meningitis and promote BBB disruption (46–49). Similarly, previous studies have shown that the GBS hemolytic pigment facilitates the penetration of GBS into the BBB and promotes proinflammatory responses in microvascular endothelial cells (21, 38). Altogether, these previous observations are in-line with our results presented here, showing that reduced dissemination of bacteria into mouse brain tissue upon infection with the Δ*lytS* mutant is accompanied by lower productions of proinflammatory cytokines (Fig. 5).

Using a mouse model of vaginal colonization, we also observed that *lytS* deficiency altered the capacity of GBS to stably establish, proliferate, and persist in the genital tract. While WT bacteria showed stable and persisting colonization in the genital tract of mice over 21 days of infection, the Δ*lytS* mutant isolate was cleared from day 14 post infection, and no viable bacteria were detectable in the genital tract on day 21 post infection. This effect could possibly be due to the reduced ability of *lytS-*deficient GBS to adhere to epithelial cells, invade the genital tract, and produce biofilms (Fig. S2). Previous studies have shown that biofilm formation could enhance the ability of GBS to colonize its host and induce invasive disease (50–53). Biofilm production is thought to be closely related to bacterial autolysis, on the basis that extracellular DNA released following cell lysis contributes to biofilm formation (54–56). As such, reduced autolysis observed in the *lytS* mutant strain lacking LytSR may be associated with its reduced capacity for biofilm production.

To the best of our knowledge, our study is the first to report that the LytSR two-component regulatory system plays a key role in GBS virulence and pathogenesis. Our result further suggests that the importance of LytSR is due to its effect on cell adhesion, biofilm production, colonization, and invasiveness. In other species, the LytSR system senses changes in membrane potential as well as extracellular metabolic signals, such as the presence of oxygen, pyruvate, or glucose, to regulate expression of a holin and potentially hundreds of other genes (28, 57). Further studies of gene regulation both *in vitro* and *in vivo* are needed to characterize how LytSR TCS affects gene expression in *S. agalactiae* during the course of infection and to understand whether the altered pathogenesis is resulting from direct regulation of known virulence factors by LytSR or whether novel mechanisms are at play. This may pave the way toward the finding of novel and more efficient strategies to prevent GBS colonization and invasive disease, particularly in susceptible populations.

## MATERIALS AND METHODS

### Bacterial strains and growth conditions

*S. agalactiae* strain HQ199 is a serotype III CC-17 clinical isolate isolated from the vaginal tract of a pregnant woman during an antenatal screening at the Liverpool Women’s Hospital. The isogenic MB-HQ199Δ*lytS* mutant was constructed as described further. GBS was routinely grown on blood agar base medium or in Todd-Hewitt broth at 37°C. For growth assay in nutritive medium, a 2-mL culture volume containing 200 CFU of GBS was grown in THB supplemented with 20% fetal bovine serum in 24-well U-bottom microplates (Greiner). Bacterial growth was monitored every 15 min for a period of 24 hours by spectrophotometry measuring the optical density at 600 nm (OD_600_) using a FLUOstar Omega microplate reader (BMG Labtech). For blood survival assay, 300 µL of heparinized human blood was inoculated with 50 CFU of GBS and incubated at 37°C at 180 rpm. At specified time points, 100 µL of the mixture was serially diluted, and dilutions were plated onto Todd-Hewitt agar medium and incubated at 37°C overnight. Viable bacteria were enumerated and expressed as CFU/mL blood. Photographs of bacterial strains grown on sheep BAB medium were captured with a Nikon D500 camera with a 16–80-mm lens.

### Construction of Δ*lytS*-deficient mutant

The isogenic *lytS* knockout mutant in *S. agalactiae* strain HQ199 was constructed by replacing *lytS* with the *aad9* cassette (conferring resistance to spectinomycin) through allelic replacement. Briefly, the *aad9* cassette and the flanking regions of *lytS* (~1,000 bp each) were amplified by PCR. The PCR products were stitched together in a second round of PCR, generating the ∆*lytS::aad9* deletion fragment, which was then ligated into pHY304 vector, generating pHY304-∆*lytS::aad9* plasmid, in which the deletion fragment provided a mean of selection of transformants. pHY304-∆*lytS::aad9* was transformed into competent GBS cells. Transformants were recovered on Todd-Hewitt agar medium supplemented with spectinomycin (Sigma; 200 µg/mL). The authenticity of *lytS* deletion in the selected transformant MB-HQ199Δ*lytS* was verified by PCR and Sanger sequencing.

### Hemolytic activity assay

Bacteria grown in THB at 37°C to an OD_600_ = 0.4 were centrifuged at 3,000 × *g* for 10 min, washed with 1× phosphate-buffered saline (PBS), and resuspended in 1 mL PBS. In a 96-well U-bottom microplate, 100 µL per well (10^8^ CFU) of the bacterial suspension was placed in the first well, and serial twofold dilutions in PBS were performed across the plate, each in a final volume = 100 µL. An equal volume of 4% fresh sheep red blood cells (TCS Biosciences) washed once and resuspended in PBS was added to each well. The plates were sealed and incubated at 37°C for 30 min. PBS alone and 0.1% sodium dodecyl sulfate (SDS) were used as negative and positive controls for hemolysis, respectively. After incubation, the plates were centrifuged at 3,000 × *g* for 10 min to pellet unlysed RBCs, and 100 µL of the supernatant was transferred into a 96-well flat-bottom microplates. Hemoglobin release was determined measuring the optical density at 420 nm (OD_420_), and percent hemolysis for the GBS-treated wells was determined relative to SDS-treated positive and PBS-treated negative controls. Three experiments were performed in triplicate.

### Autolysis assay

Triton X-100-induced autolysis assay was performed as described previously with the following modifications (58, 59). Briefly, bacteria grown overnight in THB at 37°C to an OD_600_ = 1 were centrifuged at 3,600 × *g* for 10 min. Bacterial pellets were resuspended in PBS and adjusted to an OD_600_ = 1 in 1 mL PBS containing 0.1% Triton X-100 (Sigma). The initial OD_600_ was measured (*T*_0_). Bacterial suspensions were then incubated at 37°C with shaking (180 rpm), and the OD_600_ was recorded every 15 min for a period of 2 hours. Autolysis was determined as the percent of the initial OD_600_ reading at *T*_0_. Three experiments were performed in triplicate.

### Biofilm assay

Biofilm production was determined by crystal violet (CV) staining for measuring attached biofilm as described previously by Patras et al. (58). Bacteria grown in THB at 37°C overnight were centrifuged and resuspended in PBS. Cultures were adjusted at an OD_600_ = 0.1, and 1 mL of each culture was distributed into 24-well flat-bottom plates that were incubated at 37°C for 24 hours. After incubation, wells were washed twice with 200 µL PBS to remove planktonic non-adherent cells. Wells were dried for 1 hour at room temperature prior to staining with 200 µL of 0.25% CV for 45 min at 55°C. Wells were then washed three times with dH_2_O, and 200 µL of 95% ethanol was added to wells before incubation for 30 min at room temperature. The OD_600_ was measured, and wells containing 95% ethanol were used as blank controls. Three experiments were performed in triplicate.

### Adhesion and invasion assays

Bacterial adhesion and invasion were tested by culturing human vaginal epithelial VK2/E6E7 cells in Dulbecco modified Eagle medium (DMEM) supplemented with 10% FBS and 1% penicillin (10,000 U/mL) and 1% streptomycin (10 mg/mL) to >90% confluency in 24-well plates at 37°C in 5% CO_2_. Then, 1 × 10^7^ bacteria in 1 mL DMEM were incubated at 37°C in 5% CO_2_ for 60 min for adhesion or 2 hours for invasion. After incubation, wells were washed three times with PBS. Adherence was tested by trypsinizing cells with 100 µL 0.025% trypsin-0.53 mM EDTA, adding 900 µL PBS, and plating samples serially diluted in PBS on BAB medium for determination of the numbers of CFU/well. Invasion assay mixtures were further incubated for 2 hours with 5 µg penicillin and 100 µg gentamicin at 37°C and 5% CO_2_. Wells were washed three times, and cells were trypsinized with 200 µL 0.025% trypsin-0.53 mM EDTA. Cells were lysed with 400 µL 0.1% Triton X-100, and samples were plated on BAB medium to determine the numbers of CFU/well. Three experiments were performed in triplicate.

### Mouse models of GBS vaginal colonization and sepsis

This study was conducted in strict accordance with the guidelines outlined by the UK Home Office under the Project License Number PB6DE83DA. All animal experimentations were performed at the Biomedical Services Unit, University of Liverpool and approved by the University of Liverpool Animal Welfare and Ethical Review Body.

*In vivo* experiments were performed using female BALB/c mice (5–7 weeks old) obtained from Charles River Laboratories (Kent, UK). Upon delivery, mice were allowed to acclimatize for 7 days prior to use.

For vaginal colonization, mice were synchronized into the estrus cycle by intraperitoneal administration of 17β-estradiol hormone 1 day prior to intravaginal infection with 1 × 10^6^ CFU of GBS in 10 µL. At days 1, 3, 7, 14, and 21 post infection, mice were euthanized, and vaginal, cervix, and uterus tissues were collected and placed into a sterile Bijou container containing 2 mL PBS and mechanically homogenized for ~5 s using an IKA Disperser (T 10 basic ULTRA-TURRAX, IKA, Germany). Samples were serially diluted in PBS, plated on BAB medium before incubation at 37°C overnight. CFU/mL of tissue were then enumerated.

For sepsis infection, mice were intravenously infected with 1 × 10^8^ CFU of GBS, and they were monitored every 2–3 hours for physical signs of disease using a standard scoring system (60). Mice were humanely euthanized at predefined time points, i.e., 0.5, 6, 24, and 32 hours post infection or when they were scored “2 + lethargic,” i.e., minimal movement in the absence of application of finger pressure (for survival assays). Following euthanasia, blood, lung, spleen, kidney, and brain tissues were collected. All tissues, except blood, were placed into a sterile Bijou container containing 2 mL PBS and processed as described above to enumerate the number of CFU/mL of tissue. Blood samples collected by tail bleeding at the predefined time points or cardiac puncture following euthanasia in survival assays were serially diluted in PBS and plated on BAB medium. CFU/mL were then enumerated.

### Cytokines analysis

Blood samples collected by tail bleeding were placed into a sterile tube and immediately centrifuged at 12,000 × *g* for 5 min. Serum of mice was then carefully collected and placed in a sterile tube before storage at −20°C until use. Quantification of cytokines/chemokines IL-1β, IL-2, IL-6, IL-17A/F, MIP-3α, KC/GRO, and TNF-α was performed using a U-Plex cytokine assay (Meso Scale Discovery, Rockville, Md) according to the manufacturer’s instructions.

### Statistical analysis

Statistical analysis was performed using GraphPad Prism 10 (GraphPad Software, Inc., San Diego, CA). Differences were determined using the unpaired *t* test, the Mann-Whitney *U* test, and the log-rank (Mantel-Cox) test.

## Data Availability

The genome sequence of *S. agalactiae* strain HQ199 used in this study has been deposited with links to BioProject accession number PRJNA1145887 in the NCBI BioProject database (https://www.ncbi.nlm.nih.gov/bioproject/).

## AFTER EPUB

This article was published on 14 October 2024 with an error in the corresponding author list. The corresponding authors were corrected in the current version, posted on 05 November 2024.

## SUPPLEMENTAL MATERIAL

Figure S1 spectrum.01970-24-s0001.tiff lytS deficiency prevents autolysis in GBS.

Figure S2 spectrum.01970-24-s0002.tiff lytS deficiency decreases GBS adhesion and invasion on epithelial cells and biofilm formation in vitro.

ASM does not own the copyrights to Supplemental Material that may be linked to, or accessed through, an article. The authors have granted ASM a non-exclusive, world-wide license to publish the Supplemental Material files. Please contact the corresponding author directly for reuse.
