# Supplementary figures and images for: The Streptococcus agalactiae LytSR two-component regulatory system promotes vaginal colonization and virulence in vivo
Source: Microbiol Spectr. 2024 Oct 14;12(11):e01970-24. doi: 10.1128/spectrum.01970-24 (PMC11537067; doi:10.1128/spectrum.01970-24)

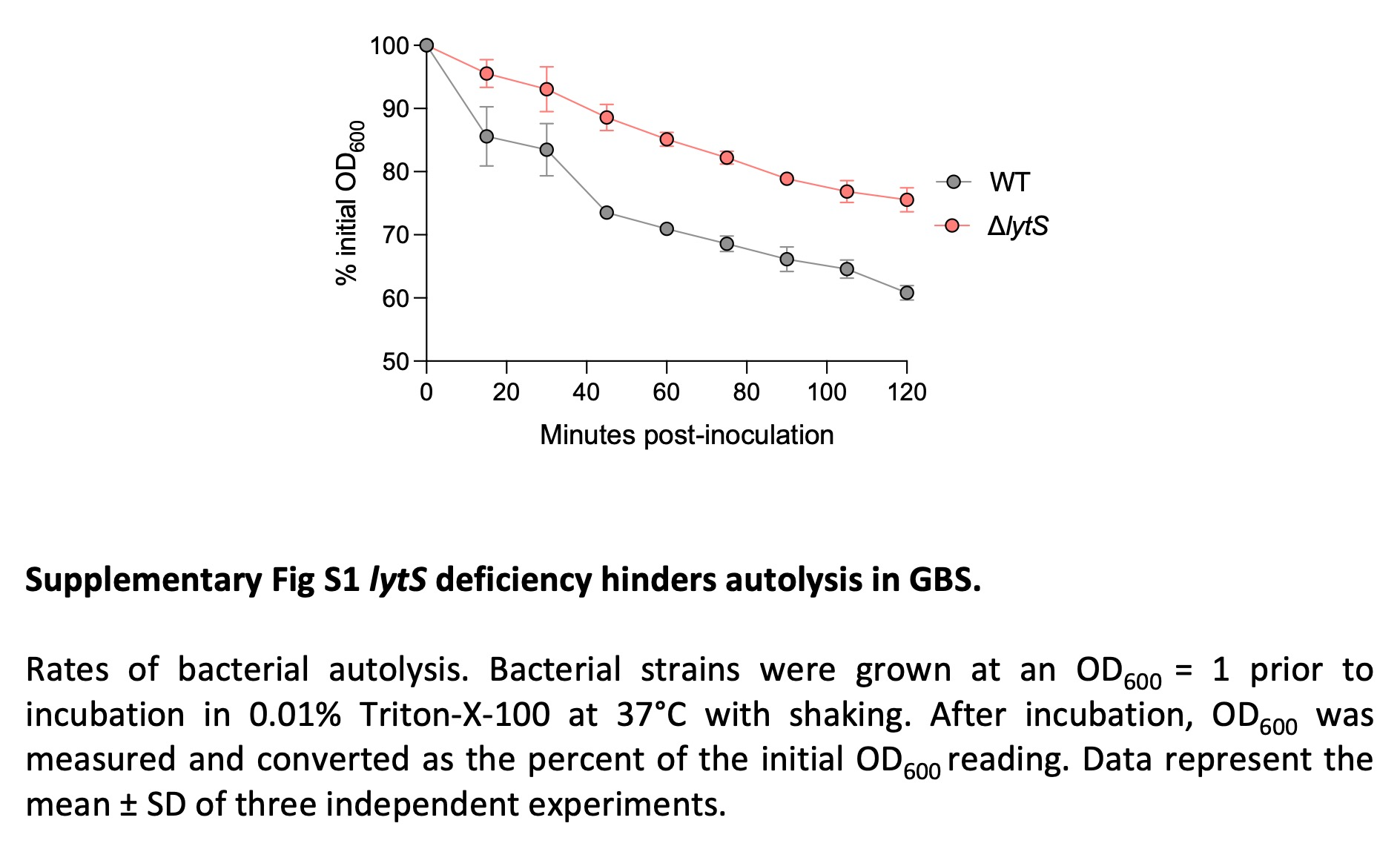

Supplement: Figure S1 — lytS deficiency prevents autolysis in GBS. [file spectrum.01970-24-s0001.tiff]

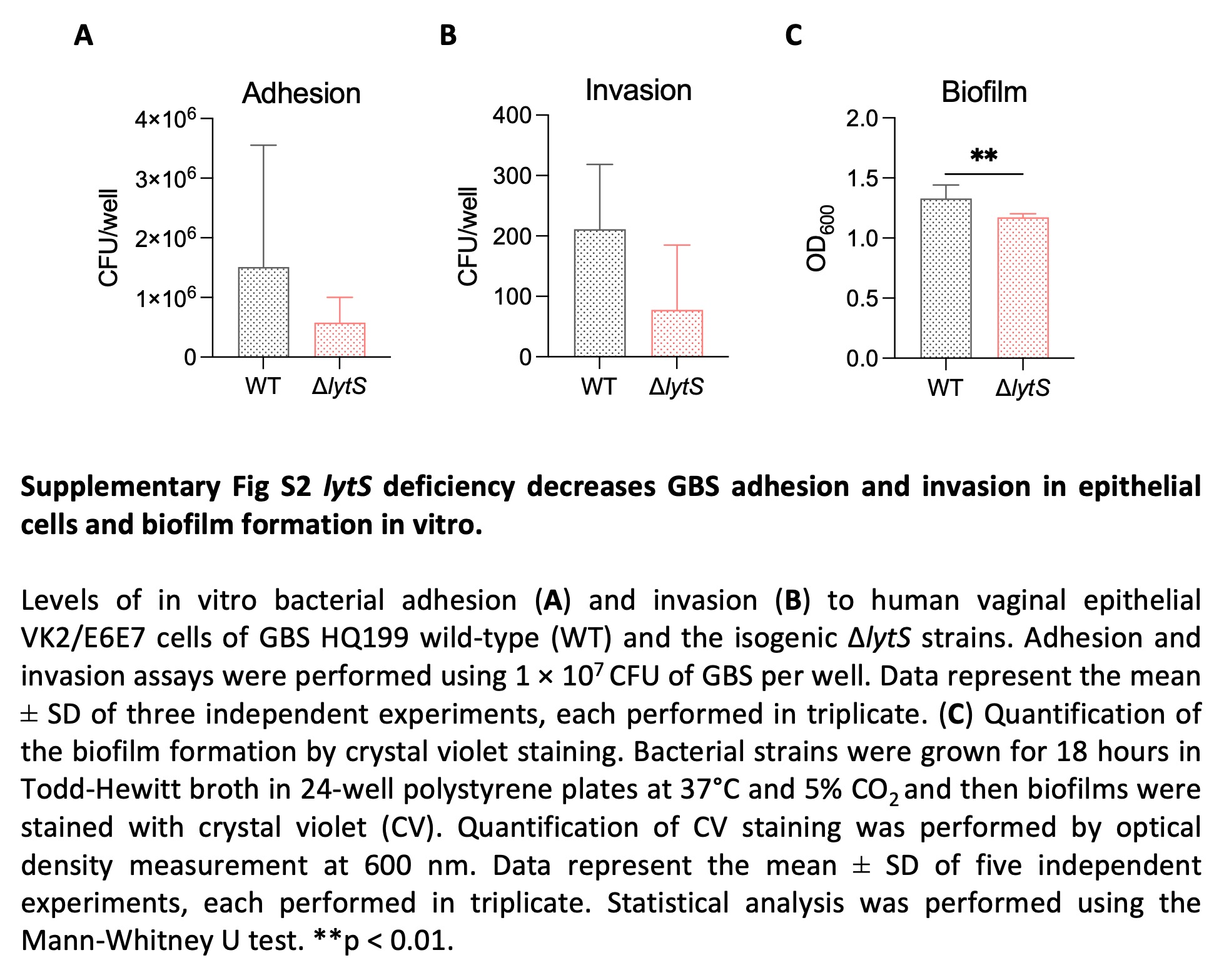

Supplement: Figure S2 — lytS deficiency decreases GBS adhesion and invasion on epithelial cells and biofilm formation in vitro. [file spectrum.01970-24-s0002.tiff]
